# Clinical efficacy and safety of thread-embedding acupuncture for treatment of the sequelae of Bell's palsy: A protocol for a patient-assessor blinded, randomized, controlled, parallel clinical trial

**DOI:** 10.1097/MD.0000000000014508

**Published:** 2019-02-15

**Authors:** Bonhyuk Goo, Seong-Mok Jeong, Jong-Uk Kim, Yeon-Cheol Park, Byung-Kwan Seo, Yong-Hyeon Baek, Tae-Han Yook, Sang-Soo Nam

**Affiliations:** a Department of Acupuncture & Moxibustion, Kyung Hee University Hospital at Gangdong, 892, Dongnam-ro, Gangdong-gu, Seoul; b Department of Clinical Korean Medicine, Graduate School, Kyung Hee University, 26, Kyungheedae-ro, Dongdaemun-gu, Seoul; c Department of Acupuncture & Moxibustion Medicine, Korean Medicine Hospital of Woosuk University, 46, Eoeun-ro, Wansan-gu, Jeonju, Republic of Korea.

**Keywords:** Bell's palsy, facial disability index, facial paralysis, sequelae, thread-embedding acupuncture

## Abstract

**Background:**

The sequelae of Bell's palsy cause critical problem in facial appearance, as well as social and psychological problems in the patient's life. The aim of the present study is to establish clinical evidence of thread-embedding acupuncture (TEA) in the treatment of sequelae of Bell's palsy.

**Method/Design:**

This is a patient-assessor blinded, randomized, sham-controlled trial with two parallel arms. Fifty-six patients aged 19–65 years, who have experienced sequelae of Bell's palsy for >3 months, will be recruited and screened using the eligibility criteria. After screening, they will be randomly allocated to a TEA group or a sham TEA (STEA) group. Both groups will receive TEA or STEA treatment on ten predefined acupoints once a week for 8 weeks. Additionally, both groups will receive the same acupuncture treatment twice a week for 8 weeks as a concurrent treatment. Changes in the Facial Disability Index over 8 weeks will be assessed as the primary outcome. Furthermore, the House-Brackmann Grade, Facial Nerve Grading System 2.0, Sunnybrook Facial Grading System, facial stiffness score, lip mobility score, and treatment satisfaction score will be measured and analyzed as secondary outcomes. All outcomes will be assessed at baseline and at 4 and 8 weeks after screening.

**Discussion:**

The results from this trial will help establish clinical evidence regarding the efficacy and safety of TEA in the treatment of patients with sequelae of Bell's palsy.

**Trial registration number:**

KCT0002557 (Clinical Research Information Service of the Republic of Korea).

## 1 Introduction

Bell's palsy, also termed idiopathic facial paralysis, is one of the most common types of peripheral facial paralysis, and the specific cause is unknown.^[1]^ Weakness of the facial muscles is the main symptom of Bell's palsy, and is accompanied by posterior auricular pain, dry eyes, hyperlacrimation, taste disorder, dry mouth, or hyperacusis.^[2]^ The severity of the symptoms varies among patients, and approximately 29% of patients are known to experience sequelae; 12% experience mild sequelae, 13% experience moderate sequelae, and 4% experience severe sequelae.^[3]^

The sequelae of Bell's palsy including synkinesis, contracture, spasm, and crocodile tear syndrome typically occur approximately 3–6 months from the onset and persist for an extended period or permanently.^[4–6]^ Patients with sequelae of Bell's palsy exhibit facial asymmetry, deformities, and abnormal movements, and complain of subjective stiffness. In a society with a high level of interest in appearance, these symptoms severely degrade patient quality of life by causing psychological or social problems.^[7,8]^

Thread-embedding acupuncture (TEA) is a novel type of acupuncture that involves the insertion and embedding of absorbable foreign substances, such as catgut or polydioxanone, into acupoints using needles. Medical thread preserves the effect of traditional acupuncture for an extended period via the mechanical and chemical stimulations of the thread.^[9,10]^ TEA has been widely used for the treatment of musculoskeletal disease,^[11,12]^ obesity,^[13]^ and cosmetic purposes, particularly for reducing facial wrinkles and improving skin elasticity.^[14,15]^

In addition to cosmetic usage, TEA has been used for therapeutic purposes for acute facial paralysis and its sequelae. Kim et al reported the adjunctive efficacy of TEA to the standard treatment of Bell's palsy within 30 days of onset.^[16]^ Ding et al reported that TEA was more effective than traditional acupuncture for the treatment of intractable facial paralysis when applied >2 months after onset.^[17]^ The clinical research of the sequelae of facial paralysis is limited to a case series,^[18]^ and the amount of evidence for TEA is not sufficient. However, because the safety and effect of TEA on facial connective tissue have been already investigated in many studies,^[19,20]^ a well-designed clinical trial will be necessary to provide further evidence and expand the therapeutic options for the sequelae of facial paralysis.

In this trial, we will compare TEA with a sham control in a rigorously designed, full-scale, randomized, controlled trial protocol to establish clinical evidence with regard to the efficacy and safety of TEA in the treatment of the sequelae of Bell's palsy.

## 2 Methods

### 2.1 Trial design

This clinical study is a patient-assessor blinded, randomized, sham-controlled trial with two parallel arms (1:1 ratio). The trial will take place at the Facial Palsy Center of Kyung Hee University Hospital at Gangdong. The efficacy and safety of TEA in patients with sequelae of Bell's palsy will be evaluated by comparison with sham TEA (STEA).

The protocol of this study has been approved by the Institutional Review Board (IRB) (KHNMCOH 2017-05-001). The trial has also been registered at the Clinical Research Information Service of the Republic of Korea (Registration Number: KCT0002557). All research procedures comply with Korean Good Clinical Practice (KGCP) and the Declaration of Helsinki. The methodological details of TEA were established in accordance with the revised Standards for Reporting Interventions in Clinical Trials of Acupuncture (STRICTA).^[21]^ The items of protocol refer to the Standard Protocol Items: Recommendations for Interventional Trials (SPIRIT).^[22]^

### 2.2 Participants

Individuals of both sexes fulfilling the following inclusion criteria will be included in this study:

(1) age 18–65 years
(2) a diagnosis of Bell's palsy made ≥3 months prior to screening
(3) Facial Disability Index (FDI) <80 on the social aspect and <70 on the physical aspect
(4) ability to communicate adequately with the researcher and respond to the questionnaire in writing
(5) willingness to sign a form stating that they will not receive any treatment other than the prescribed treatment within the treatment period, and
(6) willingness to participate in the study after providing written informed consent.

Participants with the following characteristics will be excluded:

(1) a history of receiving any other treatment that may affect the study outcomes for 2 months prior to the screening (oral administration of steroids or antiviral drugs, surgical history for treatment of facial palsy such as facial nerve compression, or facial TEA);
(2) history of hypersensitivity to TEA or severe keloid;
(3) Ramsay-Hunt syndrome;
(4) bilateral facial nerve palsy or recurrent facial nerve palsy (more than two occurrences);
(5) secondary facial nerve palsy from multiple neuritis, tumor invading the temporal bone, trauma, brain contusion, or stroke;
(6) chronic otitis media, temporomandibular fracture, or history of ear surgery;
(7) inappropriate condition for TEA administration due to skin disease or hemostatic disorder (prothrombin time international normalized ratio [PT INR] >2.0 or taking anticoagulant);
(8) liver function abnormality (aspartate transaminase [AST] or alanine transaminase [ALT] levels greater than twofold the normal level);
(9) renal function abnormality (serum creatinine or blood urea nitrogen [BUN] levels greater than twofold the normal level);
(10) pregnancy or breastfeeding;
(11) mental illness resulting in inability to comply with the clinical trial protocol; or
(12) any other condition rendering the individual unsuitable for inclusion in the trial, as determined by the principle investigator (PI).

### 2.3 Procedure

**Figure 1:**
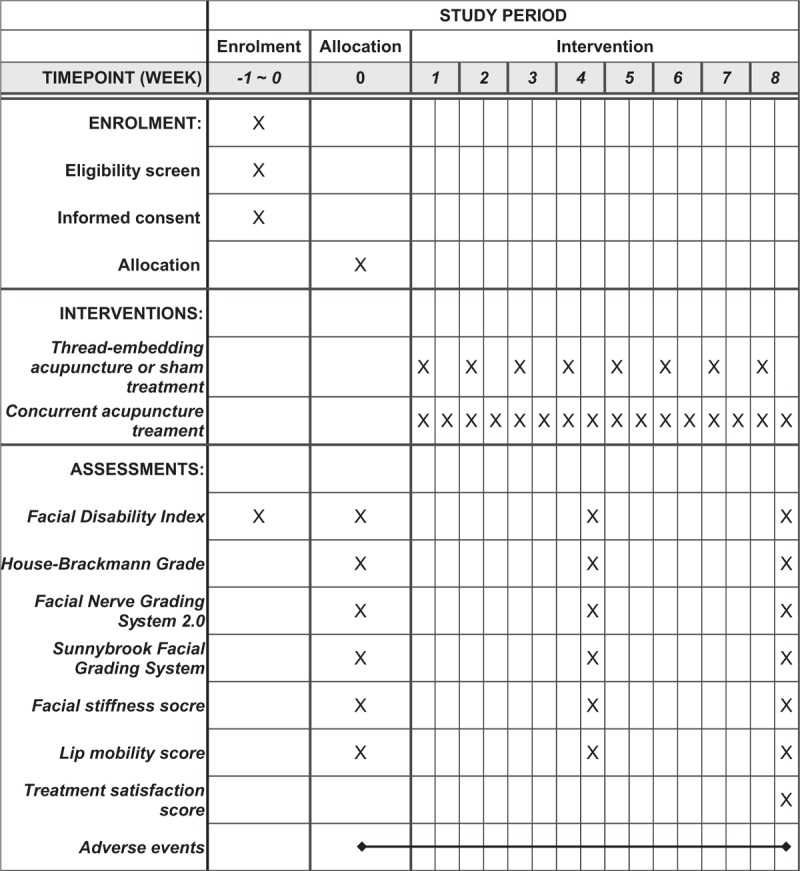
Standard Protocol Items: Recommendations for Interventional Trials (SPIRIT) figure.

Fifty-six patients with sequelae of Bell's palsy will be recruited at Kyung Hee University Hospital at Gangdong. They will be provided with detailed information regarding the study, and only those who participate voluntarily will be included in the study. Those who agree to participate and sign the informed consent form will be screened for eligibility. If they meet the eligibility criteria, the participants will be randomly allocated to the TEA group or the STEA group. After random allocation, 16 visit sessions for treatment and assessment will be conducted according to an 8-week appointed schedule (Fig. 1).

During the trial period, participants who meet the following criteria will be excluded from the study:

(1) taking medication or receiving additional treatment that is expected to affect the outcome of the study for the purpose of treating the sequelae of Bell's palsy,
(2) withdrawal of consent for study participation because the participant does not wish to continue,
(3) missing more than 5 of 16 visit sessions,
(4) occurrence of a serious adverse event (AE),
(5) critical protocol violation such as violation of eligibility criteria,
(6) decision of the PI that the participant should be removed from the trial.

### 2.4 Interventions

In both groups, TEA or STEA will be administered once a week for 8 weeks using a 29 G, 40-mm TEA needle (Hyundae Meditech, Wonju, South Korea) on 10 predefined acupoints selected by an expert group of Korean medical doctors according to STRICTA (Table 1). The selection of acupoints and details of the procedure have been determined by a consensus of clinical experts and modified from those used in previous studies.^[18,23]^ All therapeutic procedures will be performed by qualified acupuncture specialists.

**Table 1:**
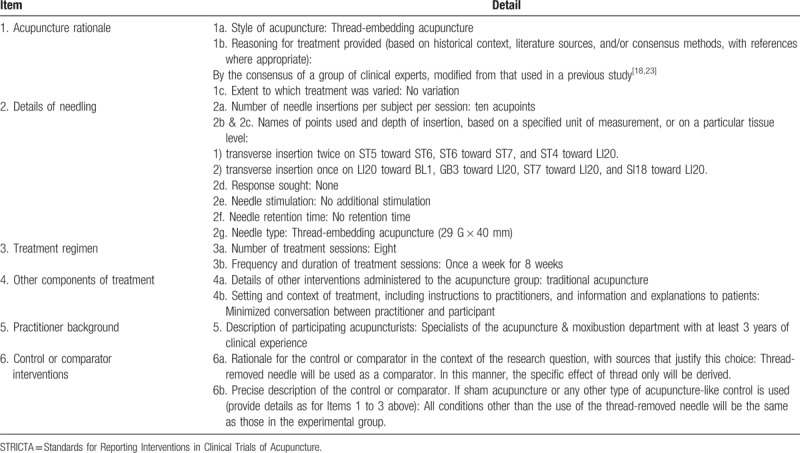
Details of the thread-embedding acupuncture treatment using the STRICTA 2010 checklist.

After covering the patient's eyes and sterilizing the skin, practitioners will conduct the intervention procedure. TEA will be transversely inserted 4 cm from one acupoint toward another acupoint in the layer termed the superficial musculoaponeurotic system,^[24]^ and only the needle will be removed while leaving the thread in the face. For the sham control comparison, thread-removed needles will be used in the STEA group instead of normal TEA in the TEA group. The thread removal procedure will be performed aseptically and discreetly for prevention of infection and patient-blinding. The practitioners will minimize the conversation during the interventions, aside from that essential to the procedure, in order to prevent bias.

### 2.5 Concurrent treatment

As a concurrent treatment, both groups will receive the same traditional needle acupuncture treatment twice a week for 8 weeks. One of the two acupuncture treatments will be planned on the same day as the intervention. Hence, acupuncture treatment will be performed immediately after TEA or STEA. For the acupuncture treatment, a 0.20 × 30 mm disposable acupuncture needle (Dongbang Medical, Seongnam, Korea) will be inserted at a depth of 5 mm and will be retained for 30 min on the following 28 acupoints: GV26, CV24, and both sides of ST3, ST4, ST6, ST7, LI20, BL2, GB14, TE23, GB1, TE17, LI4, and LR3.

Other than acupuncture treatment, any treatment that may affect the results will be prohibited. Medications that the participants have been taking from 4 weeks before the trial will be allowed at the discretion of the investigators depending on whether they affect the symptoms. All information regarding medications administered to the participants should be recorded in the case report form (CRF).

### 2.6 Outcomes

The primary outcome is a change in the FDI score. The secondary outcomes will be the House-Brackmann Grade (HB Grade), Facial Nerve Grading System 2.0 (FNGS 2.0), Sunnybrook Facial Grading System (SFGS), facial stiffness score, lip mobility score, and treatment satisfaction score. Additionally, all AEs will be recorded at each visit. All assessments will be performed by independent researchers who are not involved in the scheduled intervention.

#### 2.6.1 Primary outcome measure

##### 2.6.1.1 FDI

The FDI, which consists of ten questions including five questions regarding physical function and five questions regarding social function and well-being, is a self-reported questionnaire for assessment of disability in patients with facial nerve disorder. The FDI score will be measured at a pre-trial screening and at weeks 0 (baseline), 4, and 8 (primary end point). The change from baseline at 8 weeks will be compared between the groups as the primary outcome.

#### 2.6.2 Secondary outcome measures

##### 2.6.2.1 HB grade

The severity of facial palsy will be assessed using the HB Grade at weeks 0, 4, and 8. The Grade of I (normal function) to VI (total paralysis) is evaluated according to facial function at rest and with effort.^[25]^

##### 2.6.2.2 FNGS 2.0

Facial nerve function will be assessed using the FNGS 2.0 at weeks 0, 4, and 8. The face is divided in four regions of the brow, eye, nasolabial fold, and oral commissure; then a score of1 (normal movement) to 6 (no movement) is provided for movement of each part. Additionally, secondary movement including synkinesis and contracture is scored from 0 (none) to 3 (disfiguring synkinesis; severe contracture). Summing the scores from each region and secondary movement produces a final score of 4 to 24, which is converted to a grade of I to VI (4, I; 5–9, II; 10–14, III; 15–19, IV; 20–23, IV; 24, VI).^[26]^

##### 2.6.2.3 SFGS

Facial nerve function will also be assessed using the SFGS at weeks 0, 4, and 8. The SFGS consists of three parts: resting symmetry in three regions (eye, cheek, and mouth), symmetry of voluntary movement, and synkinesis in five expressions (forehead wrinkle, gentle eye closure, open mouth smile, snarl, and lip pucker). The scores from each part are weighted as follows: the resting symmetry score is multiplied by five, and the voluntary movement score is multiplied by four. Finally, the composite score is derived by subtracting the resting symmetry score and synkinesis score from the voluntary movement score.^[27]^

##### 2.6.2.4 Facial stiffness score

The discomfort from facial stiffness will be assessed using the facial stiffness score at weeks 0, 4, and 8. Participants will be asked “To what extent is the discomfort in your life due to the stiffness of your face?” and rate their experienced discomfort on a six-point scale (0, no discomfort; 5, the worst discomfort they can imagine).

##### 2.6.2.5 Lip mobility score

Lip mobility will be assessed using the lip-length index (LL-index) and snout index (S-index) at weeks 0, 4, and 8. The LL-index is calculated via the percentage change in lip length between grinning and resting. The S-index is calculated via the percentage change in lip length between puckering and resting. The lip length is measured by the intercommissural distance.^[28]^

##### 2.6.2.6 Treatment satisfaction score

The satisfaction with the treatment will be assessed with three questions using a 10-point scale at week 8. Participants will be asked three questions regarding their satisfaction with the treatment, intent to receive further treatment in the future, and likelihood of recommending the treatment to other people. The participants will then score each question from 1 to 10 (1, very unsatisfied; 10, very satisfied).

### 2.7 Sample size

The sample size was calculated on the basis of a previous similar study,^[29]^ as well as the advice of an expert group. With a 0.05 significance level, 90% power, 10% drop out rate, and 1:1 ratio, we determined, using the formula below, that the adequate sample size in each group is 28 participants (σ = 23.30 and *d* = 21.54): 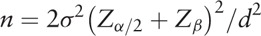


### 2.8 Randomization and allocation concealment

A total 56 participants will be randomly allocated to the TEA group or the STEA group according to a block randomization procedure with a 1:1 ratio and block size of four. The randomization sequence will be generated by an independent statistician using the SAS (SAS Institute INC., Cary, NC, USA). Random codes will then be sealed in opaque envelopes and managed by a clinical research coordinator (CRC). After participation in the trial is confirmed via screening, the CRC will open the envelope and assign the participant to the allocated group according to a random code. To conceal the allocation until the end of the study, information with regard to the allocation will be recorded in a separate log and provided only to those researchers who are carrying out the intervention.

### 2.9 Blinding

To achieve blinding of patients and assessors, only the CRC will handle the allocation information and provide restricted information to each researcher in accordance with their roles. The researchers conducting the assessment will be blinded to the allocation, and will only ask simple questions that are essential to the completion of the CRF. Participants will listen to an explanation that they will be treated using one of the two interventions: TEA or STEA. Each participant will be treated at a different time to prevent any exchange of information, and his/her eyes will be covered with a blindfold to prevent them from observing the procedure. Practitioners will minimize their conversation with the participants. An independent statistician will analyze the research data and will not be provided with information regarding the allocation. Blinding may be removed on a case-by-case basis only when the PI determines that it is essential for the safety of the patient, such as in medical emergency situations.

### 2.10 Statistical methods

The data will be corrected using the “last observation carried forward” method and then analyzed using the “intention-to-treat” analysis. The independent t-test or the Mann-Whitney U test for continuous variables and the Chi-square test or Fisher's exact test for categorical variables will be used to compare differences in general characteristics between the groups. As a primary outcome, changes in the FDI from baseline to the end of the 8-week treatment will be compared between the groups using the independent t-test. As secondary outcomes, changes in HB Grade, FNGE 2.0, SFGS, facial stiffness score, lip mobility score, and treatment satisfaction score will be also analyzed in the same way. All statistical analyses will be performed using PASW statistics 18, and the statistical significance level will be set at 0.05 (two-sided).

### 2.11 Data collection and management

The data from this trial will be collected in the CRF and cross-checked by two independent researchers. Changes in the CRF should be recorded by authorized researchers only with the date, reason, and signature. All data and documents obtained during the study period will be confidentially managed, and all output from this trial should protect anonymity. After the study is completed, all documents will be discarded or preserved for a certain period of time in accordance with management standards of the IRB.

### 2.12 Safety

To monitor the safety of the intervention and prepare for the occurrence of AEs, the following laboratory tests will be carried out at the pre-screening point and at week 8 when the all treatment is completed: complete blood count (white blood cells, red blood cells, hemoglobin, hematocrit, and platelets), liver function test (AST, ALT, alkaline phosphatase, and total bilirubin), renal function test (BUN and creatinine), erythrocyte sedimentation rate, and C-reactive protein. PT INR will be carried out if participants have hemostatic disorders or take anticoagulants. For women of childbearing age, the possibility of pregnancy should be excluded by a urine human chorionic gonadotropin test using a stick-type pregnancy test, and education of the need for medically acceptable contraception during the study period should be provided.

At each visit, the researchers will measure blood pressure, pulse rate, and body temperature, and assess the occurrence of AEs and changes in the medications being taken. Information regarding expected AEs, as well as a contact number, will be provided to the participants along with the informed consent form before the pre-trial screening. If AEs do occur, the PI will evaluate the severity of the incident, as well as its relation to the interventions, and provide proper examination and treatment in accordance with the compensation rules. The progress of all AEs will be recorded in the CRF and managed in accordance with the standard operation protocol of the IRB and the regulation of KGCP.

### 2.13 Quality control

To maintain the quality of the trial, the study procedure and documents will be periodically monitored in accordance with KGCP.

## 3 Discussion

This trial will be conducted in a randomized, sham-controlled setting in order to investigate the therapeutic effect and safety of TEA on the sequelae of Bell's palsy. TEA is typically used for cosmetic purposes such as improvement of facial wrinkles and laxity.^[23,30]^ Recently, it has been used clinically for facial paralysis in the acute and sequelae stages. Although several studies have reported the clinical effect of TEA on sequelae of facial paralysis,^[18]^ there have been no reliable, high quality clinical studies conducted to confirm the clinical benefit of TEA. Therefore, in this study, we will verify the clinical efficacy of TEA on the sequelae of Bell's palsy using a sham-control comparison and various outcome measurements.

TEA is defined as a type of acupuncture that induces therapeutic effects via the combination of acupuncture stimulation and mechanical and chemical stimulation of thread by inserting a needle with thread and leaving the thread in the body. The thread types include metal materials and animal tissue such as catgut; however, recently, thread made from polydioxanone, which is safe and absorbed with low tissue reactivity, has been used for TEA.^[15,31]^ The embedded thread affects the increase in tensile strength and stimulation of myoblast formation in connective tissue, and these mechanisms are considered to be the basis for the clinical application of this treatment.^[20]^

STEA, which will be used as a comparison in this trial, will be applied to determine the effect of specific elements of the intervention. Because the penetration or transverse insertion acupuncture technique between two acupoints has been widely used for the treatment of facial paralysis,^[32,33]^ the effect of the thread itself, excluding the elements of needle insertion, can be determined by including or excluding threads. To minimize the bias of comparison, group allocation will be blinded to participants, assessors, and statisticians.

Symptoms of the sequelae of Bell's palsy typically manifest as synkinesis, contracture, spasm, and crocodile tear syndrome, and rarely manifest as reduction of tearing, taste disorder, and hearing impairment.^[34]^ The clinical features of the sequelae of Bell's palsy manifest as complex aspects combined with problems in facial appearance, subjective stiffness, emotion, and social activity.^[35]^ Therefore, recovery from the sequelae of Bell's palsy will be evaluated via indicators of various perspectives in this trial. The physical functions that affect the patient's daily activity and social functions will be assessed via FDI, and visible problems in facial appearance and expression will be assessed by the HB Grade, FNGS 2.0, SFGS, and lip mobility score. Subjective discomfort and recovery will be assessed by the facial stiffness and treatment satisfaction scores.

The results of this study will provide clinical evidence of the changes in the comprehensive assessment of the sequelae of Bell's palsy over a period of 8weeks by comparing TEA to a sham-control treatment. These findings will aid clinicians’ understanding of TEA and highlight the treatment as a therapeutic option for patients with sequelae of Bell's palsy.

## Author contributions

**Conceptualization:** Yeon-Cheol Park, Byung-Kwan Seo, Yong-Hyeon Baek.

**Funding acquisition:** Tae-Han Yook, Sang-Soo Nam.

**Methodology:** Bonhyuk Goo, Yeon-Cheol Park, Byung-Kwan Seo, Yong-Hyeon Baek, Sang-Soo Nam.

**Project administration:** Bonhyuk Goo, Seong-Mok Jeong, Yeon-Cheol Park, Sang-Soo Nam.

**Supervision:** Yeon-Cheol Park, Byung-Kwan Seo, Tae-Han Yook, Sang-Soo Nam.

**Validation:** Jong-Uk Kim, Yeon-Cheol Park, Byung-Kwan Seo, Yong-Hyeon Baek, Tae-Han Yook, Sang-Soo Nam.

**Writing – original draft:** Bonhyuk Goo.

**Writing – review & editing:** Bonhyuk Goo, Jong-Uk Kim, Yeon-Cheol Park, Byung-Kwan Seo, Yong-Hyeon Baek, Tae-Han Yook, Sang-Soo Nam.
